# Tablet-Based Cognitive and Eye Movement Measures as Accessible Tools for Schizophrenia Assessment: Multisite Usability Study

**DOI:** 10.2196/56668

**Published:** 2024-05-30

**Authors:** Kentaro Morita, Kenichiro Miura, Atsuhito Toyomaki, Manabu Makinodan, Kazutaka Ohi, Naoki Hashimoto, Yuka Yasuda, Takako Mitsudo, Fumihiro Higuchi, Shusuke Numata, Akiko Yamada, Yohei Aoki, Hiromitsu Honda, Ryo Mizui, Masato Honda, Daisuke Fujikane, Junya Matsumoto, Naomi Hasegawa, Satsuki Ito, Hisashi Akiyama, Toshiaki Onitsuka, Yoshihiro Satomura, Kiyoto Kasai, Ryota Hashimoto

**Affiliations:** 1 Department of Rehabilitation The University of Tokyo Hospital Bunkyo-ku Tokyo Japan; 2 Department of Neuropsychiatry Graduate School of Medicine The University of Tokyo Bunkyo-ku Tokyo Japan; 3 Department of Pathology of Mental Diseases National Institute of Mental Health National Center of Neurology and Psychiatry Kodaira Japan; 4 Department of Psychiatry Graduate School of Medicine, Hokkaido University Sapporo Japan; 5 Department of Psychiatry Nara Medical University Kashihara Japan; 6 Department of Psychiatry Graduate School of Medicine, Gifu University Gifu Japan; 7 Life Grow Brilliant Mental Clinic Medical Corporation Foster Kita-ku Osaka Japan; 8 Division of Clinical Research National Hospital Organization Hizen Psychiatric Center Kanzaki-gun Japan; 9 Department of Neuroscience Division of Neuropsychiatry Yamaguchi University School of Medicine Ube City Japan; 10 Department of Psychiatry Graduate School of Biomedical Science Tokushima University Tokushima Japan; 11 Department of Neuropsychiatry Graduate School of Medicine University of Kyoto Sakyo-ku Kyoto Japan; 12 Healthcare Innovation Group Future Corporation Shinagawa-ku Tokyo Japan; 13 National Hospital Organization Sakakibara Hospital Tsu Japan; 14 Center for Diversity in Medical Education and Research Graduate School of Medicine and Faculty of Medicine The University of Tokyo Bunkyo-ku Tokyo Japan

**Keywords:** schizophrenia, cognitive function, eye movement, diagnostic biomarkers, digital health tools

## Abstract

**Background:**

Schizophrenia is a complex mental disorder characterized by significant cognitive and neurobiological alterations. Impairments in cognitive function and eye movement have been known to be promising biomarkers for schizophrenia. However, cognitive assessment methods require specialized expertise. To date, data on simplified measurement tools for assessing both cognitive function and eye movement in patients with schizophrenia are lacking.

**Objective:**

This study aims to assess the efficacy of a novel tablet-based platform combining cognitive and eye movement measures for classifying schizophrenia.

**Methods:**

Forty-four patients with schizophrenia, 67 healthy controls, and 41 patients with other psychiatric diagnoses participated in this study from 10 sites across Japan. A free-viewing eye movement task and 2 cognitive assessment tools (Codebreaker task from the THINC-integrated tool and the CognitiveFunctionTest app) were used for conducting assessments in a 12.9-inch iPad Pro. We performed comparative group and logistic regression analyses for evaluating the diagnostic efficacy of the 3 measures of interest.

**Results:**

Cognitive and eye movement measures differed significantly between patients with schizophrenia and healthy controls (all 3 measures; *P<*.001). The Codebreaker task showed the highest classification effectiveness in distinguishing schizophrenia with an area under the receiver operating characteristic curve of 0.90. Combining cognitive and eye movement measures further improved accuracy with a maximum area under the receiver operating characteristic curve of 0.94. Cognitive measures were more effective in differentiating patients with schizophrenia from healthy controls, whereas eye movement measures better differentiated schizophrenia from other psychiatric conditions.

**Conclusions:**

This multisite study demonstrates the feasibility and effectiveness of a tablet-based app for assessing cognitive functioning and eye movements in patients with schizophrenia. Our results suggest the potential of tablet-based assessments of cognitive function and eye movement as simple and accessible evaluation tools, which may be useful for future clinical implementation.

## Introduction

Schizophrenia is a severe mental illness that affects neurobiological processes, leading to difficulties in social and occupational functioning [1]. Years of research have revealed multiple candidate diagnostic markers for schizophrenia. Although some of these tests are promising, to date, none of them have been approved for use in diagnostic testing for schizophrenia [2]. This lack of approved diagnostic tests is caused by multiple factors such as the heterogeneity of mental illness, insufficient knowledge about the brain mechanisms and functions underlying mental states, and effects of known (and unknown) confounding factors [2]. Large-sample research can help overcome these issues [3]. This approach enables the observation of diverse symptoms and characteristics across a broad spectrum of individuals. However, as the sample size increases, maintaining good quality and feasibly performing assessments become difficult. Among the candidate markers for schizophrenia, cognitive function impairment and eye movement characteristics are key areas of interest. These features are not only easy to measure but also offer insights into the underlying pathophysiology of the psychiatric disorder [4].

Cognitive function impairments are observed in multiple domains among patients with schizophrenia. For the evaluation of specific cognitive dysfunctions in patients with schizophrenia, standardized test batteries have been developed by the Food and Drug Administration–National Institute of Mental Health–Measurement and Treatment Research to Improve Cognition in Schizophrenia (MATRICS), such as the MATRICS Consensus Cognitive Battery and Brief Assessment of Cognition in Schizophrenia [5,6]. Furthermore, efforts have been made to shorten and simplify these assessments [7,8]. Impairments have been replicated in aspects such as processing speed, verbal memory, working memory, attention, and executive functioning [9]. The relationships between these aspects of cognitive functioning impairment are complex. However, several network analyses have shown that processing speed has high centrality within neurocognitive functioning networks [10] and is associated with everyday life skills among community-dwelling individuals with schizophrenia [11]. The Digit Symbol Substitution Test is used to measure processing speed and is commonly included in cognitive functioning assessment batteries of patients with schizophrenia [12,13]. Due to the complexities and the multifaceted nature of cognitive impairments in patients with schizophrenia, establishing objective and reliable assessment methods is necessary.

Although cognitive function impairments are not specific to schizophrenia, there may be a greater need for objective measurements of cognitive function among patients with schizophrenia than there is among patients with other mental illnesses. In patients with schizophrenia, subjective perception of cognitive dysfunction is known; some reports suggest its association with quality of life and depression [14,15]. Although some studies indicate that subjective assessments and objective evaluations of cognitive dysfunction generally align [16,17], many papers point out discrepancies between the two [15,18-20]. Despite the emphasis on the importance of objective cognitive function assessments, these assessments have not been introduced into routine care for schizophrenia [20]. Tools such as the Digit Symbol Substitution Task are valuable assets and offer a standardized approach to quantifying cognitive deficits. Therefore, simple and objective means to measure cognitive function would be useful for the treatment of schizophrenia.

Eye movement characteristics in patients with schizophrenia have been widely studied [21]; these characteristics are among the most common behavioral deficits of this disorder. Various aspects of eye movement can be observed, such as smooth pursuit, saccade control, and visual search [21]. These analyses have been replicated in numerous samples [22-25]. In addition, these characteristics have been shown to be present from prodromal stages in patients with schizophrenia [26]. However, findings regarding the specificity of these characteristics are still inconsistent [23,27].

One example measure of eye movement is scanpath length, which is a measure of the total distance covered by the eye during visual exploration. In patients with schizophrenia, the scanpath length is generally shorter than it is in healthy controls, indicating more restricted visual exploration [28]. However, the reasons for shorter scanpath length in patients with schizophrenia are still being explored [29,30]. The simplicity of this assessment method and the need for few instructions during free-viewing tasks are beneficial for searching clinically feasible biomarkers.

Okazaki et al [31] explored the potential utility of combinations of cognitive function measures and eye movement measures in discriminating between patients with schizophrenia and healthy controls. They found that 7 pairs of cognitive functioning tests and eye movement measures, particularly pairs including digit-symbol coding or symbol search, demonstrated high discrimination performance. These pairs were acquirable in 10-15 minutes, suggesting that the combination of cognitive functioning measures and eye movement measures could be a simple and less time-consuming option for studying clinical patient groups such as patients with schizophrenia.

Based on these insights, our study aims to integrate the assessment of cognitive functioning with the analysis of eye movement characteristics. We utilized a novel tablet-based app to evaluate cognitive functioning and eye movement measurements among patients with schizophrenia. We aimed to explore whether a tablet-based app could replicate previous findings from desktop-based applications in terms of Digit Symbol Substitution Test scores and free-viewing scanpath lengths among patients with schizophrenia. Furthermore, we aimed to explore the diagnostic effectiveness of these measures in distinguishing patients with schizophrenia from other patient groups.

## Methods

### Participants

Forty-eight patients with schizophrenia, 69 healthy controls, and 49 patients with other psychiatric diagnoses were recruited from 10 clinical study sites across Japan. All study sites used the same study protocol and machines. The data were collected from distinct samples among which there were no overlapping participants. All participants were of Japanese descent and had normal or corrected-to-normal vision. Healthy controls were recruited independently at each study site through local advertisements or websites; they participated in structured interviews and were excluded from the study if a current mental illness diagnosis was suspected after the interview. Patients diagnosed with psychiatric disorders, including schizophrenia, were recruited from the outpatient and inpatient departments of each study site and included in the study after diagnosis by board-certified psychiatrists based on the Diagnostic and Statistical Manual of Mental Disorders, fifth edition criteria [32]. Distinguishing between schizophrenia and other specific psychiatric disorders is beyond the scope of this study. Therefore, we created a single group of patients with psychiatric disorders other than schizophrenia. All participants older than 12 years and who provided written consent were allowed to participate in this study (if the participant was younger than18 years, the participant provided assent, and a legal guardian provided consent before participation). The data were obtained between October and December 2022.

Two healthy controls, 4 patients with schizophrenia, and 8 patients with other psychiatric diagnoses were excluded from this study based on the predefined exclusion criteria, including deviations during measurement procedures (eg, distracting noises during the test procedure or wearing masks for infection prevention, which could disrupt the calibration process), equipment failure, or a medical history of ocular disease that may affect visual acuity (eg, glaucoma). The use of vision correction such as glasses or soft contact lenses was not a criterion for exclusion. A total of 44 patients with schizophrenia, 67 healthy controls, and 41 patients with other psychiatric diagnoses were ultimately included in the analysis. The dropout rates were 8% (4/48) among patients with schizophrenia, 3% (2/69) among healthy controls, and 16% (8/49) among patients with other psychiatric diagnoses.

The patients with other psychiatric diagnoses included 3 individuals with bipolar type 1 disorder, 5 with bipolar type 2 disorder, 10 with major depressive disorder, 19 with autism spectrum disorder, 1 with epilepsy, and 3 with comorbid diagnoses (1 individual with comorbid autism spectrum disorder and adjustment disorder, 1 with comorbid major depression and social anxiety disorder, and 1 with comorbid major depressive disorder and autism spectrum disorder).

### Ethics Approval

Ethics approval was obtained after a central review process at the National Center of Neurology and Psychiatry (B2021-120) and after reviews by the ethics committees of all 10 study sites. This study was conducted in accordance with the Declaration of Helsinki. Basic demographic information, including age, sex, and years of education, was obtained from each participant in addition to the cognitive and eye movement measurements listed below.

### Tablet Machine and Tasks

Novel tablet-based cognitive and eye movement assessments were conducted using a 12.9-inch iPad Pro (fifth generation) tablet. The display has a 2732×2048 pixel resolution at 264 ppi and a refresh rate of 120 Hz. Instructions were provided via voice or text within the app, and these instructions were given by a tester (a mental health professional) whenever necessary to ensure adequate understanding. A brief explanation about the 3 tasks used in this study is provided in Table 1.

**Table 1:** Brief descriptions about the tasks conducted.

Task name	Feature	Brief explanation	Approximate duration (min)	Measure
Codebreaker	Cognitive functioning impairment	In the Codebreaker task, participants match symbols to numbers from 1 to 6 using a key. They select matching symbols for a number sequence within 2 min to score points.	2	Codebreaker score: number of correct responses
Digit Symbol Substitution Test	Cognitive functioning impairment	In the Digit Symbol Substitution Test, participants match numbers with patterns on screen, aiming for the maximum correct pairs within 2 min.	2	Digit Symbol Substitution Test score: total score (1 point per correct)
Free-viewing test	Eye movement characteristic	In the free-viewing test, participants looked at 20 photos of buildings, items, and foods for 8 seconds each.	5	Scanpath length: mean scanpath length per image

### Cognitive Measurements

Based on the results of a previous study [31], we focused on performing cognitive assessments homologous to the Digit Symbol Substitution Test. Regarding the assessment, we used the Codebreaker task from the Japanese version of the THINC-integrated tool (THINC-it) iPad/iOS version 1.261 (THINC Task Force), which is a computerized version of the Digit Symbol Substitution Test paradigm. The reliability and stability of the THINC-it have been validated [33,34]; it has also been used in patients with schizophrenia in previous studies [35].

During the Codebreaker task, the participants were given a legend that pairs numbers ranging between 1 and 6 with specific symbols at the top of the screen. The participants were then asked to correctly associate a series of symbols with their respective numbers based on this key. During the task, the participants were shown a sequence of numbers, and the corresponding symbols must be selected from a set at the bottom of the screen. The time limit was 2 minutes; correct matches related to higher scores. There were on-screen instructions before starting the task. The correct number of responses within 2 minutes was used as the Codebreaker score in the analysis.

We also used the Digit Symbol Substitution Test app CognitiveFunctionTest (version 1.0.3; Future Corporation). The participants were instructed to pair numbers with black and white patterns. The numbers were presented in the middle of the screen, and the participants selected the corresponding patterns from the bottom of the screen. The participants were instructed to correctly select as many pairs as possible within a time limit (2 minutes). During this task, instructions were given through audio-recorded guidance and a demo session. A score of 1 was assigned for every correct answer, and a score of 0 was assigned for incorrect answers. The total Digit Symbol Substitution Test score was then calculated and used for analyses. Considering both tasks, the participants were instructed to use only 1 hand and press the display with their fingers.

### Eye Movement Measurements (iPad Pro)

The acquisition of eye movements and the calculation of eye movement measures were conducted using EyeMovementTest (version 1.0.2; Future Corporation). Eye movement measurements were performed using a front-facing camera (1200 megapixel resolution). The participants faced a tablet that was placed approximately 40 cm from their face. Eye movements were collected at a frequency of 60 Hz. A 9-point calibration and validation method was used to ensure accurate measurements of the data. The free-viewing test was conducted using 20 original photographic images of buildings, everyday items, and foods. The participants were instructed to freely view each image for 8 seconds. The images were randomly presented. A gray screen with a fixation point at the center was presented before the images, and a blank gray screen was displayed after each image. Based on previous studies [22,31], we calculated the average scanpath length across the 20 images and used this value for further analyses (scanpath length).

### Statistical Analyses

The effects of demographic characteristics on Digit Symbol Substitution Test scores, Codebreaker scores, and scanpath length were first examined in healthy controls. Correlation analysis was used to examine age and years of education, and a 2-sided Mann-Whitney *U* test was used for sex. We calculated adjusted scores by using a linear model for measures that had confounding effects among participant demographics; these were used for the analysis.

Comparisons between 2 groups were performed using the Mann-Whitney *U* test. Comparisons between 3 groups were performed using the Kruskal-Wallis test with Dunn test for pairwise comparisons. *P* values for the post hoc tests were adjusted using the Benjamini-Hochberg false discovery rate procedure to adjust for multiple comparisons. Effect sizes for group comparisons were calculated using Cliff’s δ and epsilon squared (denoted as ε^2^).

The classification performance of the outcomes of interest was tested using a simple logistic regression model with leave-one-out cross validation. Receiver operating characteristic (ROC) analyses and confusion matrices were used to assess the performance of the classifier. The threshold value for each model was determined from the ROC curve, as this value could provide the best balance between sensitivity and specificity. Bootstrap resampling with 1000 iterations was used to calculate the 95% CIs for accuracy, specificity, and sensitivity. We also performed an exploratory analysis of the classification performance of the same model to distinguish patients with schizophrenia from those with other psychiatric diagnoses. The patients with schizophrenia were considered as patients in both classification analyses.

Data analyses were performed using R version 4.3.1 (R Foundation for Statistical Computing) implemented in R studio. The libraries *rstatix* (version 0.7.2) and *effsize* (version 0.8.1) were used for statistical analysis; the libraries *pROC* (version 1.18.5), *caret* (version 6.0.94), and *plyr* (version 1.8.9) were used for classification and model evaluation; and the library *ggplot2* (version 3.4.4) was used for visualization.

## Results

### Exploratory Analysis of the Potential Confounders

We first explored the effects of demographic characteristics (age, years of education, and sex) on the 3 outcomes measured by the tablet app among healthy controls. The demographic characteristics of each group are provided in Table S1 in Multimedia Appendix 1. Correlation analysis revealed that Codebreaker scores and Digit Symbol Substitution Test scores were negatively correlated with age (ρ=–0.50; *P<*.001 and ρ=–0.34; *P<*.001, respectively) and positively correlated with years of education (ρ=0.32; *P<*.001 and ρ=0.24; *P*=.049, respectively). Free viewing was not significantly correlated with age (ρ=0.18; *P*=.15) or education years (ρ=0.17; *P*=.17). The Mann-Whitney *U* test revealed that there were no significant sex differences in Codebreaker scores (*U*=467; *P*=.35), Digit Symbol Substitution Test scores (*U*=565; *P*=.75), or scanpath length (*U*=652; *P*=.16) between the groups. Based on these results, we calculated age- and years of education–adjusted values for the Codebreaker scores and Digit Symbol Substitution Test scores. Thereafter, we used these values for the following analyses (unless otherwise specified as raw scores).

### Distributions of the Measurement Values

**Figure 1:**
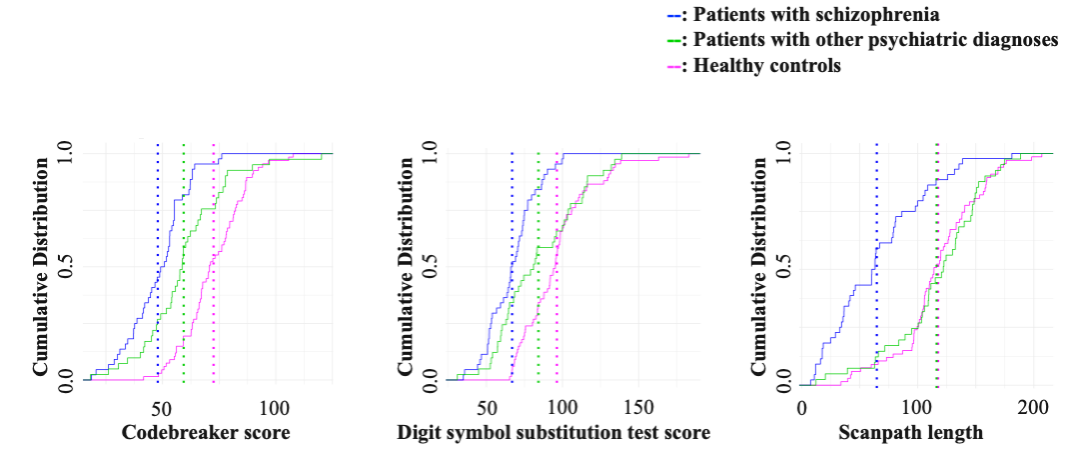
Distribution of the measured values. The cumulative distribution function for the 3 measures of interest (ie, Codebreaker score, Digit Symbol Substitution Test score, and scanpath length) are provided across the 3 groups. The dotted lines in each plot represent the mean values in each group.

We first examined the distribution of each measure in patients with schizophrenia, healthy controls, and patients with other psychiatric disorders. To visualize the distributions of each measure, we developed plots for the cumulative distribution function of each measure (Figure 1). The cumulative distribution function accumulated probabilities to indicate the likelihood of a value being at or below a certain point. Healthy controls and patients with schizophrenia differed significantly in all measures, with the Codebreaker scores having the largest difference (*P*<.001; Cliff’s δ value –0.82, 95% CI –0.90 to –0.69). Notably, the distribution of scanpath length showed overlapping patterns between healthy controls and patients with other psychiatric disorders and did not differ significantly (*P*=.92; Cliff’s δ value=–0.03, 95% CI –0.20 to 0.25). Group comparisons of each measure are provided in Table S2 in Multimedia Appendix 1.

### Classification Performance in Patients With Schizophrenia and in Healthy Controls

**Figure 2:**
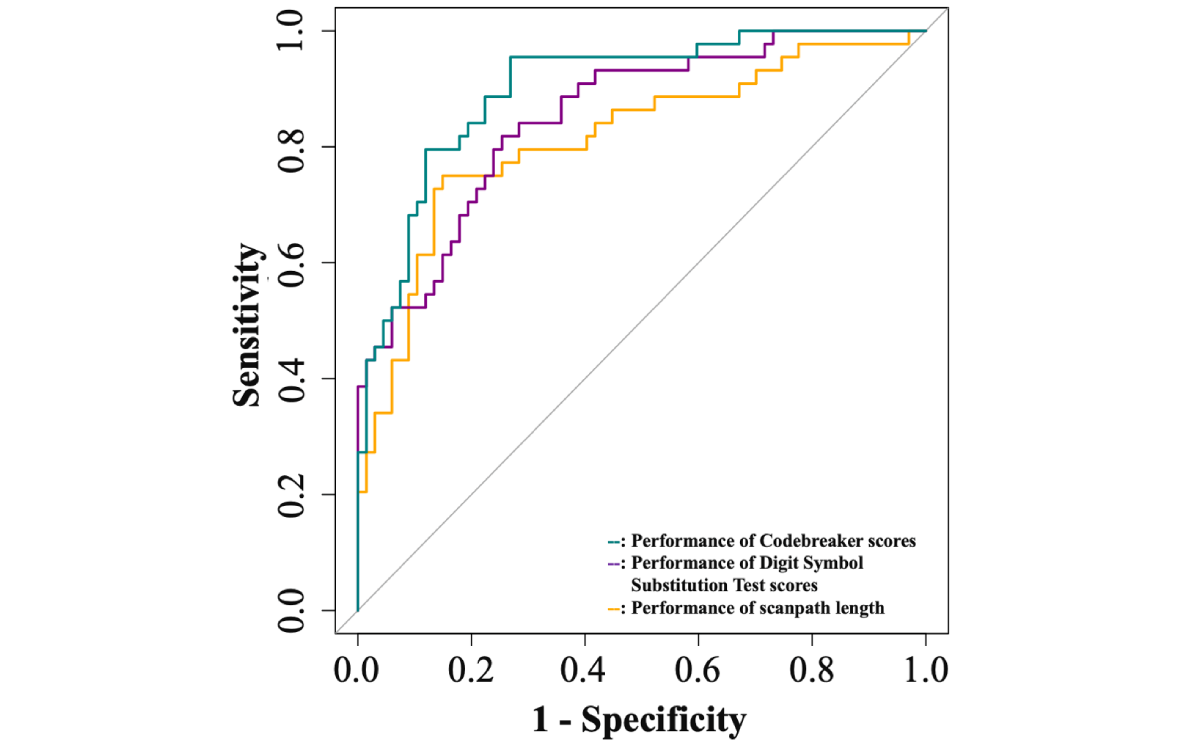
Receiver operating characteristic curves for each of the 3 outcomes of interest.

To test the diagnostic utility of the 3 measures, we classified the 2 diagnostic groups as dependent variables by using a logistic regression model and each of the 3 outcomes of interest as independent variables (Table 2). The area under the curve (AUC) was the largest for the Codebreaker score, with a value of 0.90 (95% CI 0.84-0.96). The accuracy (0.82, 95% CI 0.74-0.89) and specificity (0.95, 95% CI 0.89-1.00) were also the highest for the Codebreaker score, but the sensitivity was the highest for scanpath length (0.85, 95% CI 0.76-0.93). The ROC curves are shown in Figure 2. We also tested the classification performance of combining measures to identify the model with the best performance. The AUC was the largest for the model combining all 3 measures (AUC 0.94, 95% CI 0.90-0.98).

**Table 2:** Summary of the classification performance metrics in patients with schizophrenia versus healthy control participants.^a^

	Area under the curve (95% CI)	Accuracy (95% CI)	Sensitivity (95% CI)	Specificity (95% CI)
Codebreaker score	0.90 (0.84-0.96)	0.82 (0.74-0.89)	0.73 (0.62-0.83)	0.95 (0.89-1.00)
Digit Symbol Substitution Test score	0.85 (0.78-0.92)	0.77 (0.69-0.85)	0.75 (0.64-0.85)	0.82 (0.70-0.92)
Scanpath length	0.81 (0.73-0.90)	0.81 (0.73-0.88)	0.85 (0.76-0.93)	0.75 (0.63-0.87)
Codebreaker score + Digit Symbol Substitution Test score	0.91 (0.86-0.96)	0.85 (0.77-0.91)	0.87 (0.78-0.94)	0.82 (0.70-0.93)
Codebreaker score + scanpath length	0.93 (0.88-0.97)	0.84 (0.76-0.90)	0.78 (0.68-0.87)	0.93 (0.85-1.00)
Digit Symbol Substitution Test score + scanpath length	0.90 (0.85-0.96)	0.85 (0.77-0.91)	0.94 (0.88-0.99)	0.70 (0.55-0.83)
Codebreaker score + Digit Symbol Substitution Test score + scanpath length	0.94 (0.90-0.98)	0.85 (0.77-0.91)	0.84 (0.75-0.92)	0.86 (0.75-0.95)

^a^Logistic regression models were used to classify patients with schizophrenia and healthy controls according to each measure of interest. We implemented leave-one-out cross-validation to estimate the generalizability of the trained model. We also conducted classifications by using pairs of eye movement and cognitive functioning measures or a combination of all 3 measures.

### Classification Performance in Patients With Schizophrenia and in Patients With Other Psychiatric Diagnoses

The model made to classify patients with schizophrenia and healthy controls was also tested for classification performance in patients with schizophrenia and those with other psychiatric diagnoses (Table 3). This analysis revealed that scanpath length had the largest AUC (0.82, 95% CI 0.73-0.91), accompanied by notable accuracy (0.78, 95% CI 0.67-0.86) and sensitivity (0.83, 95% CI 0.71-0.93). Conversely, the Digit Symbol Substitution Test score showed the highest specificity (0.93, 95% CI 0.85-1.00), albeit with the lowest sensitivity (0.41, 95% CI 0.27-0.57). Table 3 also assesses the combined efficacy of eye movement and cognitive functioning measures in classification, which did not increase in this analysis.

**Table 3:** Summary of the classification performance metrics in patients with schizophrenia versus those with other psychiatric diagnoses. The models, which include individual measures and their combinations, were initially trained using data of healthy controls and patients with schizophrenia.

	Area under the curve (95% CI)	Accuracy (95% CI)	Sensitivity (95% CI)	Specificity (95% CI)
Codebreaker score	0.70 (0.59-0.81)	0.71 (0.60-0.80)	0.61 (0.46-0.76)	0.80 (0.68-0.91)
Digit Symbol Substitution Test score	0.68 (0.57-0.80)	0.68 (0.57-0.78)	0.41 (0.27-0.57)	0.93 (0.85-1.00)
Scanpath length	0.82 (0.73-0.91)	0.78 (0.67-0.86)	0.83 (0.71-0.93)	0.73 (0.59-0.85)
Codebreaker score + Digit Symbol Substitution Test score	0.72 (0.61-0.83)	0.68 (0.57-0.78)	0.37 (0.23-0.52)	0.98 (0.92-1.00)
Codebreaker score + scanpath length	0.80 (0.70-0.89)	0.75 (0.65-0.84)	0.71 (0.57-0.84)	0.80 (0.67-0.90)
Digit Symbol Substitution Test score + scanpath length	0.81 (0.72-0.90)	0.76 (0.66-0.85)	0.88 (0.77-0.97)	0.66 (0.52-0.80)
Codebreaker score + Digit Symbol Substitution Test score + scanpath length	0.80 (0.71-0.89)	0.75 (0.65-0.84)	0.76 (0.62-0.88)	0.75 (0.62-0.87)

Due to the small sample size, the same patients with schizophrenia were used in both the training and testing phases for the previous analysis. Therefore, we also conducted leave-one-out cross validation for a model trained for all participants, classifying patients with schizophrenia versus participants without schizophrenia (healthy controls and patients with other diagnoses). Similar performance was seen for codebreaker score (AUC 0.82, 95% CI 0.75-0.89) and scanpath length (AUC 0.81, 95% CI 0.73-0.89). Combining all measures highly increased the performance (AUC 0.88, 95% CI 0.83-0.94). The complete results are presented in Table S3 in Multimedia Appendix 1.

## Discussion

In this multisite study conducted in Japan, we assessed the results of tablet-based measurements of cognitive function and eye movement characteristics in patients with schizophrenia, healthy controls, and in patients with other psychiatric diagnoses. All 3 measures significantly differed among patients with schizophrenia, healthy controls, and those with other psychiatric diagnoses. We also demonstrated that the individual measures had high classification performances. The Codebreaker score was the most effective single measure in distinguishing patients with schizophrenia from healthy controls. Additionally, scanpath length was the most effective single measure in distinguishing patients with schizophrenia from those with other psychiatric diagnoses. Combining all 3 measures further improved the diagnostic performance. These findings suggest that cognitive and eye movement assessments performed using tablet-based platforms could have potential utility as a novel diagnostic approach.

Using a novel tablet-based measurement, we were able to replicate the findings of Digit Symbol Substitution Task deficits and shorter scanpath lengths in participants with schizophrenia compared with healthy controls. Cliff’s δ effect sizes of the measures obtained using the tablet app were greater for the Codebreaker score (–0.82) and Digit Symbol Substitution Test score (–0.73) than that for the scanpath length (–0.66). A meta-analysis on processing speed deficits in patients with schizophrenia for Digit Symbol Substitution Test scores revealed effect sizes (Hedge *g*) ranging between –1.57 and –1.34 [36]. Regarding scanpath length, in our previous study [22], the effect size (Cohen *d*) was –1.5, and in a literature review by Beedie et al [28], the pooled effect size (Hedge *g*) calculated across 28 studies was –0.77 [28]. Therefore, the results of this study seemed to replicate the trend observed in previous studies.

A key finding of our study was the extent to which simple tablet-based assessments were able to match desktop-based tests in terms of distinguishing patients with schizophrenia from healthy controls. The AUC for scanpath length was 0.81, while the AUCs for cognitive function measures ranged between 0.85 and 0.90. These results were similar to the findings of previous studies using desktop-based eye movement measurements, which reported AUCs ranging between 0.77 and 0.89 [22,23,26], and previous studies using paper-based cognitive functioning measurements, which reported AUCs ranging between 0.88 and 0.90 [37,38]. Okazaki et al [31] found that pairing certain cognitive function measures with eye movement measures led to improved performance and robustness in distinguishing patients with schizophrenia from healthy individuals, with an average increase in the AUC of 0.10 compared with eye movement measures alone and 0.05 compared with cognitive function measures alone. Our study revealed similar results—combinations of the 3 measures of interest yielded AUC values ranging between 0.90 and 0.94. These results are promising, given the ease and simplicity of performing cognitive function and eye movement assessments using a tablet.

We further explored the differences between patients with schizophrenia and those with other psychiatric diagnoses. We found differences in both cognitive function measures and eye movement measures, with larger differences in eye movement measures. This finding is in line with that in previous studies that established diagnostic markers for differentiating between patients with schizophrenia and those without schizophrenia by using eye movement characteristics [39,40]. However, previous studies indicated mixed results of both cognitive function [41-43] and eye movement [23,40,44]. Conducting future studies on the transdiagnostic characteristics of multiple modalities is increasingly important. Moreover, mobile measurement will help this process by making studies possible by using less expensive and more accessible formats.

The usage of mobile formats for measuring cognitive function has already been reported in the field of psychiatry and elsewhere. Several studies have assessed the validity of mobile (tablet-based or smartphone-based) apps compared with paper-based studies and have shown equivalent performance [8,45-48]. One study utilized the simplicity of mobile apps to conduct a longitudinal study in which cognitive assessments were conducted outside the experimental setting and had higher completion rates in patients with schizophrenia than in the control group [49]. However, only a few case-control comparisons have been performed [49,50]. Additionally, previous studies included small sample sizes, and there is a need for studying the specific effects of using technological tools, such as accessibility and digital literacy [51].

Eye movement characteristics have not been studied using mobile devices in patients with schizophrenia. Studies [52-55] have used portable eye trackers. An old but innovative study focused on the reliability of a portable head-mounted display for tracking eye movements in patients with schizophrenia and found that it was comparable to traditional fixed-display setups [52]. Dowiasch et al [53] examined the eye movements of patients with schizophrenia in a natural, everyday setting, contrasting them with healthy controls. They identified distinct eye movement behaviors such as more frequent but shorter fixations in patients with schizophrenia. Other studies used portable devices but also required participants to fix their head during the tasks [24,54,55]. These tools are still expensive, and mobile tools such as ours are yet to be utilized in the field of psychiatry.

To the best of our knowledge, there are no studies of tablet-based or simplified measurement tools that focus on both cognitive function and eye movement assessments in patients with schizophrenia. With our tablet-based measurement tool, we were able to observe different classification features of cognitive functioning measures (Codebreaker scores and Digit Symbol Substitution Test scores) and for the eye movement measure (scanpath length). Cognitive functioning measures were more useful for differentiating patients with schizophrenia from healthy controls, while scanpath length differed more between patients with schizophrenia and those with other psychiatric diagnoses. By combining the 2 measures, we were also able to increase diagnostic performance. This is interesting, given that both cognitive function and eye movement have long been known to be affected in patients with schizophrenia [56,57]. One possibility is that differences in neurobiological substrates lead to digit symbol substitution impairments and abnormal visual searches [58,59]. Psychiatric disorders exhibit both within-disorder heterogeneity and transdiagnostic features, and recent large-scale neuroimaging studies have shown disease-specific and shared neuroanatomical alterations [60,61]. Combining different features of schizophrenia, such as combining cognitive function and eye movement measures, may be beneficial not only for disease classification but also for biotyping, which may lead to developing new treatment options in the future.

The use of tablet-based assessment tools such as the one used in this study may facilitate development and implementation of auxiliary diagnostic tools in the field of psychiatry. Conventional cognitive assessment batteries are time-consuming and require special training for implementation, and conventional eye movement measurement requires the use of expensive and complex equipment, necessitating expert setup and skilled operation for measurements. Some studies have found ways to conduct these conventional assessments in a less time-consuming way [31,37]. However, our study is novel in that it combines simplified measurements of cognitive function and eye movement, which do not require such expertise. The tablet used in our study might allow for an inexpensive, more convenient, and compact form of evaluation, combining psychological and neurophysiological findings of schizophrenia. Tablet-based tools will make such assessments more accessible and convenient for patients and clinicians alike and may lead to having more objective information to aid decision-making in the clinical setting.

This study has several limitations. One limitation is the small sample size of participants included in this study, and our research would require replication considering larger samples. Another is that 2 of the measures used in this study (the Digit Symbol Substitution Test score and scanpath length) have still to be tested for reliability and validity like the Codebreaker task [33,62]. The effects of possible confounders were also not assessed in detail. Although we employed age- and years of education–adjusted scores to account for demographic differences between the groups, this was a linear correction due to the small sample size. Further analyses of age- and years of education–matched samples may be needed. There also remains the possibility of other confounding factors such as medication usage that has not been studied or fully controlled. Several studies have shown that shorter scanpath lengths [28] and slower processing speeds [63] are present before medication usage. Additionally, the results of cognitive functioning measures, including the Codebreaker test, have been known to change longitudinally [35,64,65]. In our study, we did not assess the longitudinal robustness of our findings. Studies with larger sample sizes in various age groups and reliability and validity testing of the measures as well as addressing the limitations above would enhance the robustness and generalizability of our findings.

In this study, we successfully demonstrated the feasibility of using novel tablet-based apps to perform cognitive function and eye movement assessments among patients with schizophrenia. Our findings align with those of previous research indicating significant differences in cognitive and eye movement measures between patients with schizophrenia and healthy controls, with the key feature of being able to accomplish this using tablet-based assessments. In particular, the measurement of eye movement by using a tablet device is unique to this study. These findings suggest that assessments using such digital tools may hold the potential for use in various clinical settings, such as an auxiliary diagnostic tool to aid the decision-making process in psychiatric treatment in the future.

## Appendix

Multimedia Appendix 1 Supplementary tables.

## Abbreviations

AUC: area under the curve
MATRICS: Measurement and Treatment Research to Improve Cognition in Schizophrenia
ROC: receiver operating characteristic
THINC-it: THINC-integrated tool

## References

[1] Jauhar Sameer, Johnstone Mandy, McKenna Peter J (2022). Schizophrenia. Lancet.

[2] Abi-Dargham A, Moeller SJ, Ali F, DeLorenzo C, Domschke K, Horga G, Jutla A, Kotov R, Paulus MP, Rubio JM, Sanacora G, Veenstra-VanderWeele J, Krystal JH (2023). Candidate biomarkers in psychiatric disorders: state of the field. World Psychiatry.

[3] Onitsuka T, Hirano Y, Nemoto K, Hashimoto N, Kushima I, Koshiyama D, Koeda M, Takahashi T, Noda Y, Matsumoto J, Miura K, Nakazawa T, Hikida T, Kasai K, Ozaki N, Hashimoto R (2022). Trends in big data analyses by multicenter collaborative translational research in psychiatry. Psychiatry Clin Neurosci.

[4] Onitsuka Toshiaki, Hirano Yoji, Nakazawa Takanobu, Ichihashi Kayo, Miura Kenichiro, Inada Ken, Mitoma Ryo, Yasui-Furukori Norio, Hashimoto Ryota (2022). Toward recovery in schizophrenia: Current concepts, findings, and future research directions. Psychiatry Clin Neurosci.

[5] Kern RS, Green MF, Nuechterlein KH, Deng B (2004). NIMH-MATRICS survey on assessment of neurocognition in schizophrenia. Schizophr Res.

[6] Keefe RSE, Harvey PD, Goldberg TE, Gold JM, Walker TM, Kennel C, Hawkins K (2008). Norms and standardization of the Brief Assessment of Cognition in Schizophrenia (BACS). Schizophr Res.

[7] Lam M, Wang M, Huang W, Eng GK, Rapisarda A, Kraus M, Kang S, Keefe RSE, Lee J (2017). Establishing the brief assessment of cognition - short form. J Psychiatr Res.

[8] Atkins AS, Tseng T, Vaughan A, Twamley EW, Harvey P, Patterson T, Narasimhan M, Keefe RSE (2017). Validation of the tablet-administered Brief Assessment of Cognition (BAC App). Schizophr Res.

[9] Gebreegziabhere Y, Habatmu K, Mihretu A, Cella M, Alem A (2022). Cognitive impairment in people with schizophrenia: an umbrella review. Eur Arch Psychiatry Clin Neurosci.

[10] Karyakina M, Shmukler A (2021). Network analysis of cognitive deficit in patients with schizophrenia spectrum disorders. Schizophr Res Cogn.

[11] Galderisi S, Rucci P, Mucci A, Rossi A, Rocca P, Bertolino A, Aguglia E, Amore M, Bellomo A, Bozzatello P, Bucci P, Carpiniello B, Collantoni E, Cuomo A, Dell'Osso L, Di Fabio F, di Giannantonio M, Gibertoni D, Giordano GM, Marchesi C, Monteleone P, Oldani L, Pompili M, Roncone R, Rossi R, Siracusano A, Vita A, Zeppegno P, Maj M, Italian Network for Research on Psychoses (2020). The interplay among psychopathology, personal resources, context-related factors and real-life functioning in schizophrenia: stability in relationships after 4 years and differences in network structure between recovered and non-recovered patients. World Psychiatry.

[12] Keefe RSE, Goldberg TE, Harvey PD, Gold JM, Poe MP, Coughenour L (2004). The brief assessment of cognition in schizophrenia: reliability, sensitivity, and comparison with a standard neurocognitive battery. Schizophr Res.

[13] Nuechterlein KH, Green MF, Kern RS, Baade LE, Barch DM, Cohen JD, Essock S, Fenton WS, Frese FJ, Gold JM, Goldberg T, Heaton RK, Keefe RSE, Kraemer H, Mesholam-Gately R, Seidman LJ, Stover E, Weinberger DR, Young AS, Zalcman S, Marder SR (2008). The MATRICS Consensus Cognitive Battery, part 1: test selection, reliability, and validity. Am J Psychiatry.

[14] Paudel S, Coman D, Freudenreich O (2020). Subjective experience of cognitive difficulties as an important attribute of quality of life among individuals with schizophrenia spectrum disorders. Schizophr Res.

[15] Sellwood W, Morrison AP, Beck R, Heffernan S, Law H, Bentall RP (2013). Subjective cognitive complaints in schizophrenia: relation to antipsychotic medication dose, actual cognitive performance, insight and symptoms. PLoS One.

[16] Lecardeur L, Briand C, Prouteau A, Lalonde P, Nicole L, Lesage A, Stip E (2009). Preserved awareness of their cognitive deficits in patients with schizophrenia: convergent validity of the SSTICS. Schizophr Res.

[17] Gilleen J, Greenwood K, David AS (2011). Domains of awareness in schizophrenia. Schizophr Bull.

[18] Medalia A, Thysen J (2010). A comparison of insight into clinical symptoms versus insight into neuro-cognitive symptoms in schizophrenia. Schizophr Res.

[19] Burton CZ, Harvey PD, Patterson TL, Twamley EW (2016). Neurocognitive insight and objective cognitive functioning in schizophrenia. Schizophr Res.

[20] McCutcheon RA, Keefe RSE, McGuire PK (2023). Cognitive impairment in schizophrenia: aetiology, pathophysiology, and treatment. Mol Psychiatry.

[21] Morita K, Miura K, Kasai K, Hashimoto R (2020). Eye movement characteristics in schizophrenia: A recent update with clinical implications. Neuropsychopharmacol Rep.

[22] Morita Kentaro, Miura Kenichiro, Fujimoto M, Yamamori H, Yasuda Y, Iwase Masao, Kasai Kiyoto, Hashimoto Ryota (2017). Eye movement as a biomarker of schizophrenia: Using an integrated eye movement score. Psychiatry Clin Neurosci.

[23] St Clair D, MacLennan G, Beedie SA, Nouzová Eva, Lemmon H, Rujescu D, Benson PJ, McIntosh A, Nath M (2022). Eye movement patterns can distinguish schizophrenia from the major affective disorders and healthy control subjects. Schizophr Bull Open.

[24] Lencer R, Sprenger A, Reilly JL, McDowell JE, Rubin LH, Badner JA, Keshavan MS, Pearlson GD, Tamminga CA, Gershon ES, Clementz BA, Sweeney JA (2015). Pursuit eye movements as an intermediate phenotype across psychotic disorders: Evidence from the B-SNIP study. Schizophr Res.

[25] Radant AD, Millard SP, Braff DL, Calkins ME, Dobie DJ, Freedman R, Green MF, Greenwood TA, Gur RE, Gur RC, Lazzeroni LC, Light GA, Meichle SP, Nuechterlein KH, Olincy A, Seidman LJ, Siever LJ, Silverman JM, Stone WS, Swerdlow NR, Sugar CA, Tsuang MT, Turetsky BI, Tsuang DW (2015). Robust differences in antisaccade performance exist between COGS schizophrenia cases and controls regardless of recruitment strategies. Schizophr Res.

[26] Lyu H, St Clair D, Wu R, Benson Pj, Guo W, Wang G, Liu Y, Hu S, Zhao J (2023). Eye movement abnormalities can distinguish first-episode schizophrenia, chronic schizophrenia, and prodromal patients from healthy controls. Schizophr Bull Open Oxford Academic.

[27] Takahashi J, Hirano Y, Miura K, Morita K, Fujimoto M, Yamamori H, Yasuda Y, Kudo N, Shishido E, Okazaki K, Shiino T, Nakao T, Kasai K, Hashimoto R, Onitsuka T (2021). Eye movement abnormalities in major depressive disorder. Front Psychiatry.

[28] Beedie SA, Benson PJ, St Clair DM (2011). Atypical scanpaths in schizophrenia: evidence of a trait- or state-dependent phenomenon?. J Psychiatry Neurosci.

[29] Okada K, Miura K, Fujimoto M, Morita K, Yoshida M, Yamamori H, Yasuda Y, Iwase M, Inagaki M, Shinozaki T, Fujita I, Hashimoto R (2021). Impaired inhibition of return during free-viewing behaviour in patients with schizophrenia. Sci Rep.

[30] Yoshida M, Miura K, Fujimoto M, Yamamori H, Yasuda Y, Iwase M, Hashimoto R (2024). Visual salience is affected in participants with schizophrenia during free-viewing. Sci Rep.

[31] Okazaki K, Miura K, Matsumoto J, Hasegawa N, Fujimoto M, Yamamori H, Yasuda Y, Makinodan M, Hashimoto R (2023). Discrimination in the clinical diagnosis between patients with schizophrenia and healthy controls using eye movement and cognitive functions. Psychiatry Clin Neurosci.

[32] American Psychiatric Association (2013). Diagnostic and Statistical Manual of Mental Disorders, Fifth Edition.

[33] Harrison JE, Barry H, Baune BT, Best MW, Bowie CR, Cha DS, Culpepper L, Fossati P, Greer TL, Harmer C, Klag E, Lam RW, Lee Y, Mansur RB, Wittchen H, McIntyre RS (2018). Stability, reliability, and validity of the THINC-it screening tool for cognitive impairment in depression: A psychometric exploration in healthy volunteers. Int J Methods Psychiatr Res.

[34] McIntyre RS, Best MW, Bowie CR, Carmona NE, Cha DS, Lee Y, Subramaniapillai M, Mansur RB, Barry H, Baune BT, Culpepper L, Fossati P, Greer TL, Harmer C, Klag E, Lam RW, Wittchen H, Harrison J (2017). The THINC-Integrated Tool (THINC-it) screening assessment for cognitive dysfunction: validation in patients with major depressive disorder. J Clin Psychiatry.

[35] Szmyd JK, Lewczuk K, Teopiz KM, McIntyre RS, Wichniak A (2023). THINC-Integrated Tool (THINC-it): a brief measurement of changes in cognitive functioning and its correlation with the life quality of patients with schizophrenia and related disorders-a pilot study. Brain Sci.

[36] Knowles EEM, David AS, Reichenberg A (2010). Processing speed deficits in schizophrenia: reexamining the evidence. Am J Psychiatry.

[37] Fujino H, Sumiyoshi C, Yasuda Y, Yamamori H, Fujimoto M, Fukunaga M, Miura K, Takebayashi Y, Okada N, Isomura S, Kawano N, Toyomaki A, Kuga H, Isobe M, Oya K, Okahisa Y, Takaki M, Hashimoto N, Kato M, Onitsuka T, Ueno T, Ohnuma T, Kasai K, Ozaki N, Sumiyoshi T, Imura O, Hashimoto R, COCORO for (2017). Estimated cognitive decline in patients with schizophrenia: A multicenter study. Psychiatry Clin Neurosci.

[38] Bezdicek O, Michalec J, Kališová Lucie, Kufa T, Děchtěrenko F, Chlebovcová Miriama, Havlík Filip, Green MF, Nuechterlein KH (2020). Profile of cognitive deficits in schizophrenia and factor structure of the Czech MATRICS Consensus Cognitive Battery. Schizophr Res.

[39] Kojima T, Matsushima E, Ohta K, Toru M, Han YH, Shen YC, Moussaoui D, David I, Sato K, Yamashita I, Kathmann N, Hippius H, Thavundayil JX, Lal S, Vasavan Nair NP, Potkin SG, Prilipko L (2001). Stability of exploratory eye movements as a marker of schizophrenia--a WHO multi-center study. World Health Organization. Schizophr Res.

[40] Suzuki M, Takahashi S, Matsushima E, Tsunoda M, Kurachi M, Okada T, Hayashi T, Ishii Y, Morita K, Maeda H, Katayama S, Kawahara R, Otsuka T, Hirayasu Y, Sekine M, Okubo Y, Motoshita M, Ohta K, Uchiyama M, Kojima T (2009). Exploratory eye movement dysfunction as a discriminator for schizophrenia : a large sample study using a newly developed digital computerized system. Eur Arch Psychiatry Clin Neurosci.

[41] Goldberg T E, Gold J M, Greenberg R, Griffin S, Schulz S C, Pickar D, Kleinman J E, Weinberger D R (1993). Contrasts between patients with affective disorders and patients with schizophrenia on a neuropsychological test battery. Am J Psychiatry.

[42] Lewandowski KE, Sperry SH, Cohen BM, Ongür D (2014). Cognitive variability in psychotic disorders: a cross-diagnostic cluster analysis. Psychol Med.

[43] Sumiyoshi C, Ohi K, Fujino H, Yamamori H, Fujimoto M, Yasuda Y, Uno Y, Takahashi J, Morita K, Katsuki A, Yamamoto M, Okahisa Y, Sata A, Katsumoto E, Koeda M, Hirano Y, Nakataki M, Matsumoto J, Miura K, Hashimoto N, Makinodan M, Takahashi T, Nemoto K, Kishimoto T, Suzuki M, Sumiyoshi T, Hashimoto R (2022). Transdiagnostic comparisons of intellectual abilities and work outcome in patients with mental disorders: multicentre study. BJPsych Open.

[44] Bestelmeyer PEG, Tatler BW, Phillips LH, Fraser G, Benson PJ, St Clair D (2006). Global visual scanning abnormalities in schizophrenia and bipolar disorder. Schizophr Res.

[45] Pratt DN, Luther L, Kinney KS, Osborne KJ, Corlett PR, Powers AR, Woods SW, Gold JM, Schiffman J, Ellman LM, Strauss GP, Walker EF, Zinbarg R, Waltz JA, Silverstein SM, Mittal VA (2023). Comparing a computerized digit symbol test to a pen-and-paper classic. Schizophr Bull Open.

[46] Tang S, Chen I, Chiang H, Wu C, Hsueh I, Yu W, Hsieh C (2018). A comparison between the original and Tablet-based Symbol Digit Modalities Test in patients with schizophrenia: Test-retest agreement, random measurement error, practice effect, and ecological validity. Psychiatry Res.

[47] Cubillos C, Rienzo A (2023). Digital cognitive assessment tests for older adults: systematic literature review. JMIR Ment Health.

[48] McIntyre RS, Lipsitz O, Rodrigues NB, Subramaniapillai M, Nasri F, Lee Y, Fehnert B, King J, Chrones L, Kratiuk K, Uddin S, Rosenblat JD, Mansur RB, McCue M (2022). An app-based digit symbol substitution test for assessment of cognitive deficits in adults with major depressive disorder: evaluation study. JMIR Ment Health.

[49] Liu G, Henson P, Keshavan M, Pekka-Onnela J, Torous J (2019). Assessing the potential of longitudinal smartphone based cognitive assessment in schizophrenia: A naturalistic pilot study. Schizophr Res Cogn.

[50] Cornelis C, De Picker LJ, Hulstijn W, Dumont G, Timmers M, Janssens L, Sabbe BGC, Morrens M (2014). Preserved learning during the symbol-digit substitution test in patients with schizophrenia, age-matched controls, and elderly. Front Psychiatry.

[51] Lavigne KM, Sauvé Geneviève, Raucher-Chéné Delphine, Guimond S, Lecomte T, Bowie CR, Menon M, Lal S, Woodward TS, Bodnar MD, Lepage M (2022). Remote cognitive assessment in severe mental illness: a scoping review. Schizophrenia (Heidelb).

[52] Hong LE, Avila MT, Wonodi I, McMahon RP, Thaker GK (2005). Reliability of a portable head-mounted eye tracking instrument for schizophrenia research. Behav Res Methods.

[53] Dowiasch S, Backasch B, Einhäuser Wolfgang, Leube D, Kircher T, Bremmer F (2016). Eye movements of patients with schizophrenia in a natural environment. Eur Arch Psychiatry Clin Neurosci.

[54] Delerue C, Boucart M (2013). Visual exploration and action processing in schizophrenia. Cogn Neuropsychiatry.

[55] Obyedkov I, Skuhareuskaya M, Skugarevsky O, Obyedkov V, Buslauski P, Skuhareuskaya T, Waszkiewicz N (2019). Saccadic eye movements in different dimensions of schizophrenia and in clinical high-risk state for psychosis. BMC Psychiatry.

[56] Dickinson D, Ramsey ME, Gold JM (2007). Overlooking the obvious: a meta-analytic comparison of digit symbol coding tasks and other cognitive measures in schizophrenia. Arch Gen Psychiatry.

[57] Phillips ML, David AS (1998). Abnormal visual scan paths: a psychophysiological marker of delusions in schizophrenia. Schizophr Res.

[58] Koshiyama D, Fukunaga M, Okada N, Yamashita F, Yamamori H, Yasuda Y, Fujimoto M, Ohi K, Fujino H, Watanabe Y, Kasai K, Hashimoto R (2018). Role of subcortical structures on cognitive and social function in schizophrenia. Sci Rep.

[59] Morita K, Miura K, Fujimoto M, Yamamori H, Yasuda Y, Kudo N, Azechi H, Okada N, Koshiyama D, Shiino T, Fukunaga M, Watanabe Y, Ikeda M, Kasai K, Hashimoto R (2019). Eye-movement characteristics of schizophrenia and their association with cortical thickness. Psychiatry Clin Neurosci.

[60] Matsumoto J, Fukunaga M, Miura K, Nemoto K, Okada N, Hashimoto N, Morita K, Koshiyama D, Ohi K, Takahashi T, Koeda M, Yamamori H, Fujimoto M, Yasuda Y, Ito S, Yamazaki R, Hasegawa N, Narita H, Yokoyama S, Mishima R, Miyata J, Kobayashi Y, Sasabayashi D, Harada K, Yamamoto M, Hirano Y, Itahashi T, Nakataki M, Hashimoto R, Tha KK, Koike S, Matsubara T, Okada G, Yoshimura R, Abe O, van Erp TGM, Turner JA, Jahanshad N, Thompson PM, Onitsuka T, Watanabe Y, Matsuo K, Yamasue H, Okamoto Y, Suzuki M, Ozaki N, Kasai K, Hashimoto R (2023). Cerebral cortical structural alteration patterns across four major psychiatric disorders in 5549 individuals. Mol Psychiatry.

[61] Okada N, Fukunaga M, Miura K, Nemoto K, Matsumoto J, Hashimoto N, Kiyota M, Morita K, Koshiyama D, Ohi K, Takahashi T, Koeda M, Yamamori H, Fujimoto M, Yasuda Y, Hasegawa N, Narita H, Yokoyama S, Mishima R, Kawashima T, Kobayashi Y, Sasabayashi D, Harada K, Yamamoto M, Hirano Y, Itahashi T, Nakataki M, Hashimoto R, Tha KK, Koike S, Matsubara T, Okada G, van Erp TGM, Jahanshad N, Yoshimura R, Abe O, Onitsuka T, Watanabe Y, Matsuo K, Yamasue H, Okamoto Y, Suzuki M, Turner JA, Thompson PM, Ozaki N, Kasai K, Hashimoto R (2023). Subcortical volumetric alterations in four major psychiatric disorders: a mega-analysis study of 5604 subjects and a volumetric data-driven approach for classification. Mol Psychiatry.

[62] Dalby M, Annas P, Harrison JE, 23andMe Research Team (2022). Further validation of the THINC-it tool and extension of the normative data set in a study of n = 10.019 typical controls. Int J Methods Psychiatr Res.

[63] Peng X, Hei G, Yang Y, Liu C, Xiao J, Long Y, Huang J, Zhao J, Wu R (2021). The association between cognitive deficits and clinical characteristic in first-episode drug naïve patients with schizophrenia. Front Psychiatry.

[64] Lennertz L, An der Heiden W, Kronacher R, Schulze-Rauschenbach S, Maier W, Häfner Heinz, Wagner M (2016). Smaller than expected cognitive deficits in schizophrenia patients from the population-representative ABC catchment cohort. Eur Arch Psychiatry Clin Neurosci.

[65] McIntyre RS, Subramaniapillai M, Park C, Zuckerman H, Cao B, Lee Y, Iacobucci M, Nasri F, Fus D, Bowie CR, Tran T, Rosenblat JD, Mansur RB (2020). The THINC-it Tool for cognitive assessment and measurement in major depressive disorder: sensitivity to change. Front Psychiatry.

