# Supplementary material for: Tablet-Based Cognitive and Eye Movement Measures as Accessible Tools for Schizophrenia Assessment: Multisite Usability Study
Source: JMIR Ment Health. 2024 May 30;11:e56668. doi: 10.2196/56668 (PMC11176872; doi:10.2196/56668)
Supplement: Multimedia Appendix 1 [file mental_v11i1e56668_app1.docx]

# Tablet-Based Cognitive and Eye Movement Measures as Accessible Tools for Schizophrenia Assessment: Multisite Usability Study

### **Authors**

Kentaro Morita, Kenichiro Miura, Atsuhito Toyomaki, Manabu Makinodan, Kazutaka Ohi, Naoki Hashimoto, Yuka Yasuda, Takako Mitsudo, Fumihiro Higuchi, Shusuke Numata, Akiko Yamada, Yohei Aoki, Hiromitsu Honda, Ryo Mizui, Masato Honda, Daisuke Fujikane, Junya Matsumoto, Naomi Hasegawa, Satsuki Ito, Hisashi Akiyama, Toshiaki Onitsuka, Yoshihiro Satomura, Kiyoto Kasai, Ryota Hashimoto

**Supplementary materials**

Table S1

Table S2

Table S3

**Table S1** The demographic characteristics of the three subject groups are presented.

|  | **SZ (n=44)** | **HC (n=67)** | **OT (n=41)** |
| --- | --- | --- | --- |
| Age | 43.9 (10.9) | 35.3 (11.6) | 36.7 (13.9) |
| Years of education | 13.6 (2.1) | 15.8 (2.3) | 14.3 (2.2) |
| Sex (male/female) | 23/21 (52%) | 40/27 (60%) | 21/20 (51%) |

This table presents the demographic characteristics of patients with schizophrenia (SZ), healthy controls (HCs), and those with other psychiatric disorders (OTs). The table includes the means (standard deviations) for age and years of education and the number of participants of each sex, with the percentage of male individuals in parentheses.

**Table S2** Comparison of the three measures of interest between the patients with schizophrenia, healthy controls, and those with other psychiatric disorders is listed.

|  | **Subject group** | **Mean (SD)** | **[95% CI]** | **Kruskal‒Wallis Test**^a^ | **Pairwise comparisons** | | |
| --- | --- | --- | --- | --- | --- | --- | --- |
|  |  |  |  |  | **SZ vs. HC** | **SZ vs. OT** | **OT vs. HC** |
|  | SZ(n=44) | 47.9(13.1) | [43.9, 51.9] | H(2)=57 | δ=-0.82 | δ=-0.40 | δ=-0.48 |
| **Codebreaker score** | HC(n=67) | 72.5(13.7) | [69.1, 75.8] | p=3.44 × 10^-13^ | [-0.90, -0.69] | [-0.60, -0.15] | [-0.65, -0.27] |
|  | OT(n=41) | 59.3(19.0) | [53.3, 65.3] | *ε*2=0.0025 | p=2.41 × 10^-13^ | p=3.01 × 10^-3^ | p=9.70 × 10^-5^ |
|  | SZ(n=44) | 67.0(16.0) | [62.1, 71.9] | H(2)=39 | δ=-0.73 | δ=-0.37 | δ=-0.27 |
| **Digit Symbol Substitution Test score** | HC(n=67) | 96.4(23.2) | [90.7, 102.1] | p=2.98 × 10^-9^ | [-0.84, -0.57] | [-0.57, -0.12] | [-0.49, -0.02] |
|  | OT(n=41) | 84.3(27.1) | [75.7, 92.8] | *ε2*=0.0017 | p=1.13 × 10^-9^ | p=2.41 × 10^-3^ | p=9.74 × 10^-3^ |
|  | SZ(n=44) | 64.8(41.4) | [52.2, 77.4] | H(2)=39 | δ=-0.66 | δ=-0.64 | δ=-0.03 |
| **Scanpath length** | HC(n=67) | 117.6(36.3) | [108.7, 126.4] | p=3.23 × 10^-9^ | [-0.79, -0.46] | [-0.78, -0.42] | [-0.20, 0.25] |
|  | OT(n=41) | 116.3(40.2) | [103.6, 129.0] | *ε2*=0.0017 | p=3.16 × 10^-8^ | p=3.76 × 10^-7^ | p=9.17 × 10^-1^ |

This table presents statistical comparisons of Codebreaker score, Digit Symbol Substitution Test score, and Scanpath length among the three groups: patients with schizophrenia (SZ), healthy controls (HC), and those with other psychiatric disorders (OT). The table includes the mean, standard deviation (SD), 95% confidence interval (95% CI), epsilon squared effect size (ε^2^), Cliff’s δ effect sizes (with 95% CIs), and *p* values, with asterisks indicating statistical significance at *p* < 0.05. The Kruskal‒Wallis test indicated significant differences between these groups for all the measurements. Post hoc analysis using Dunn’s tests with false discovery rate adjustments highlighted key contrasts: healthy controls and patients with schizophrenia differed significantly in all measures (Codebreaker score: p value: 2.41 × 10^−13^; Cliff’s δ value -0.82 [95% CI:-0.90, -0.69]); patients with schizophrenia and patients with other psychiatric disorders also showed notable differences across all metrics (largest difference in Scanpath length: p value: 3.76 × 10^−7^; Cliff’s δ value -0.64 [95% CI: -0.78, -0.42]). Scanpath length did not significantly differ between the healthy control group and the patients with other psychiatric disorders (*p* value=0.917; Cliff’s δ value=-0.03 [95% CI=-0.20, 0.25]).

**Table S3** Classification performance in patients with schizophrenia and those without schizophrenia is summarized.

|  | **AUC** | **Accuracy** | **Sensitivity** | **Specificity** |
| --- | --- | --- | --- | --- |
| **Codebreaker score** | 0.82 [0.75, 0.89] | 0.78 [0.70, 0.84] | 0.77 [0.68, 0.84] | 0.80 [0.67, 0.90] |
| **Digit Symbol Substitution Test score** | 0.78 [0.71, 0.86] | 0.70 [0.62, 0.78] | 0.66 [0.58, 0.75] | 0.82 [0.71, 0.93] |
| **Scanpath length** | 0.81 [0.73, 0.89] | 0.81 [0.74, 0.87] | 0.83 [0.77, 0.90] | 0.75 [0.61, 0.88] |
| **Codebreaker score  + Digit Symbol Substitution Test score** | 0.83 [0.77, 0.90] | 0.78 [0.70, 0.84] | 0.81 [0.73, 0.88] | 0.70 [0.57, 0.84] |
| **Codebreaker score  + Scanpath length** | 0.88 [0.82, 0.93] | 0.81 [0.74, 0.87] | 0.80 [0.72, 0.87] | 0.84 [0.71, 0.94] |
| **Digit Symbol Substitution Test score + Scanpath length** | 0.86 [0.80, 0.92] | 0.79 [0.72, 0.85] | 0.78 [0.69, 0.85] | 0.82 [0.69, 0.93] |
| **Codebreaker score  + Digit Symbol Substitution Test score + Scanpath length** | 0.88 [0.83, 0.94] | 0.84 [0.77, 0.90] | 0.89 [0.83, 0.94] | 0.73 [0.58, 0.86] |

This table shows the logistic regression model used to differentiate patients with schizophrenia from those without schizophrenia (healthy controls and patients with other psychiatric diagnoses). We implemented leave-one-out cross-validation (LOOCV) to estimate the generalizability of the trained model. Performance metrics such as the area under the curve (AUC), accuracy, sensitivity, and specificity are provided, along with 95% confidence intervals for each measure.
